# Mutational Spectrum of *LDLR* and *PCSK9* Genes Identified in Iranian Patients With Premature Coronary Artery Disease and Familial Hypercholesterolemia

**DOI:** 10.3389/fgene.2021.625959

**Published:** 2021-02-11

**Authors:** Arman Moradi, Majid Maleki, Zahra Ghaemmaghami, Zahra Khajali, Feridoun Noohi, Maryam Hosseini Moghadam, Samira Kalyinia, Seyed Javad Mowla, Nabil G. Seidah, Mahshid Malakootian

**Affiliations:** ^1^Department of Molecular Genetics, Faculty of Biological Sciences, Tarbiat Modares University, Tehran, Iran; ^2^Cardiogenetic Research Center, Rajaie Cardiovascular Medical and Research Center, Iran University of Medical Sciences, Tehran, Iran; ^3^Laboratory of Biochemical Neuroendocrinology, Montreal Clinical Research Institute, University of Montreal, Montreal, QC, Canada

**Keywords:** pre-mature CAD, Familial Hypercholesterolemia, loss-of-function, LDLR, PCSK9 (proprotein convertase subtilisin kexin type 9)

## Abstract

Familial hypercholesterolemia (FH) is a common, yet underdiagnosed, genetic disorder characterized by lifelong elevated low-density lipoprotein cholesterol levels, which can increase the risk of early-onset coronary artery disease (CAD). In the present study, we screened the nucleotide variations of the *LDLR and PCSK9* genes, as well as a part of the *APOB* gene, in Iranian patients with FH and premature CAD to find the genetic cause of the disorder. Fifteen unrelated individuals with a clinical diagnosis of FH and premature CAD were recruited. Direct DNA sequencing was applied to screen the whole coding exons and exon–intron boundaries of the *LDLR* and *PCSK9* genes and the main parts of their introns, together with exon 26 of the *APOB* gene. The pathogenicity of the identified mutations was investigated via either segregation analyses in the family or *in silico* predictive software. Six different point mutations (p.Cys148Tyr, p.Cys216Tyr, p.Cys302Trp, p.Cys338Trp, p.Leu479Gln, and p.G593Afs^∗^72) in *LDLR* and a double mutation (p.Asp172His and p.Ala53Val) in both *LDLR* and *PCSK9* genes were identified in seven families with clinically diagnosed FH (43%), whereas no pathogenic mutations were found in eight families with clinically diagnosed FH. This study is the first to identify 1 pathogenic mutation in the *LDLR* gene (c.1014C > G [p.Cys338Trp]) and to cosegregate it from the affected individual in the family. No mutations were found in the *APOB* gene, whereas several silent mutations/polymorphisms were identified in the *LDLR* and *PCSK9* genes. Genetic testing and reports on nucleotide alterations in the Iranian population are still limited. Our findings not only further confirm the significant role of FH in the incidence of premature CAD but also enlarge the spectrum of *LDLR* and *PCSK9* variations and exhibit the heterogeneity of FH in Iranians. In patients with no mutation in the examined genes, the disease could be begotten either by a polygenic cause or by gene defects occurring in other related genes and regions not targeted in this study.

## Background

Familial hypercholesterolemia (FH) is a monogenic disorder of the metabolism of low-density lipoprotein cholesterol (LDL-C) and is characterized by lifelong elevated levels of LDL particles and LDL-C arterial deposits (Khachadurian, 1964; Brown and Goldstein, 1974). Untreated patients carry the risk of early onset coronary artery disease (premature CAD) and the augmented risk of cardiovascular events (Nordestgaard et al., 2013; Cuchel et al., 2014b; Gidding et al., 2015; Krogh et al., 2016). The current prevalence of FH is estimated to be 1:311 individuals in the general population (Beheshti et al., 2020; Hu et al., 2020).

This monogenic disorder is inherited in two forms of autosomal dominant and autosomal recessive (De Castro-Orós et al., 2010; Cuchel et al., 2014a). The autosomal dominant form of FH is mostly due to the heterozygous and homozygous states of pathogenic variants in the *low-density lipoprotein receptor* (*LDLR*), *apolipoprotein B* (*APOB*), and *proprotein convertase subtilisin/kexin type 9* (*PCSK9*) genes. Between 85 and 90% of the mutations harbored in *LDLR* that result in defective LDL receptors attenuate LDL-C clearance from the blood and, consequently, raise LDL-C plasma levels (Varret and Rabès, 2012).

Likewise, mutations in the *APOB* gene weaken the binding of LDL to LDL receptors, and gain-of-function mutations in the *PCSK9* gene account for 5–15% of cases with FH (El Khoury et al., 2017; Sharifi et al., 2017). PCSK9, a serine protease, mediates the internalization and degradation of LDLR in the lysosome via binding to LDL receptors, leading to diminished LDLR recycling and impaired clearance of LDL-C particles from the plasma (Seidah, 2017). Recently, next-generation sequencing has demonstrated the FH phenotype due to occasional dominant mutations in *apolipoprotein E* (*APOE*) or *signal-transducing adaptor family member 1* (*STAP1*), as well (Nordestgaard et al., 2013; Cuchel et al., 2014b; Fouchier et al., 2014; Berberich and Hegele, 2019).

The autosomal recessive form of FH is frequently in consequence of pathogenic mutations in the *low-density lipoprotein receptor adaptor protein* (*LDLRAP*) gene (Cuchel et al., 2014a). Nevertheless, whole-exome sequencing has revealed that mutations in the *lysosomal acid lipase A* (*LIPA*), *ATP-binding cassette sub-family G member 5 and 8* (*ABCG5/8*), and *cholesterol 7 alpha-hydroxylase* (*CYP7A1*) genes phenotypically cause hypercholesterolemia similar to FH in the recessive status (Brautbar et al., 2015; Berberich and Hegele, 2019).

At present, the diagnosis of FH is frequently based on clinical phenotypes, with the most extensively utilized FH clinical criteria being the Simon Broome register (SBR) (Simon Broome Register Group., 1991) and the Dutch Lipid Clinic Network System (DLCNS) (Nordestgaard et al., 2013). In this study, we analyzed the possible mutations of the *LDLR*, *APOB*, and *PCSK9* genes in Iranian patients suffering from both FH and premature CAD.

## Materials and Methods

### Subjects

The present study enrolled 15 unrelated patients with clinically diagnosed FH and premature CAD who met the SBR and/or DLCNS criteria. The demographic characteristics, clinical features, and cholesterol levels of the study population are presented in Table 1. The SB and DLCNS criteria encompass the levels of total cholesterol (TC) and LDL-C, the presence of tendon xanthomas, and a family history of hypercholesterolemia, premature CAD, or cardiovascular events in a first- and/or second-degree relative.

**TABLE 1:** Clinical characteristics of the enrolled patients.

Patient ID	LDL-C (mg/dL)	Total cholesterol (mg/dL)	Gender	Age	Coronary artery disease	Intervention	Family history		Xanthomas	Corneal ring	Consanguine marriage	SB	DLCNS
							Hypercholes-terolemia	Coronary artery disease or myocardial infarction					
P1	240	417	F	11	Yes	CABG -AVR	Yes	MI,CAD	Yes	–	Yes	Definite	Definite
P2	276	338	F	37	Yes	–	Yes	MI,CAD	–	–	Yes	Possible	Definite
P3	203	288	F	45	Yes	–	Yes	MI	–	–	–	Possible	Definite
P4	370	458	F	40	Yes	CABG	Yes	CAD,MI	Yes	–	–	Definite	Definite
P5	95	179	F	48	Yes	CABG	Yes	CAD,MI	–	–	–	–	Possible
P6	105.6	192	M	47	Yes	CABG-AVR	Yes	CAD	–	Yes	–	Possible	Possible
P7	222	339	F	55	Yes	CABG	Yes	CAD	–	Yes	–	Definite	Definite
P8	391	461	M	24	Yes, MI	CABG-AVR	Yes	MI,CAD	Yes	Yes	Yes	Definite	Definite
P9	340	415	M	22	Yes	–	Yes	MI	Yes	–	Yes	Definite	Definite
P10	153	229	M	30	Yes	CABG	Yes	CAD	–	Yes	Yes	Definite	Definite
P11	250	358	M	32	Yes	PCI	Yes	CAD	–	–	Yes	Possible	Definite
P12	252	355	F	38	Yes	PCI	Yes	MI	–	–	–	Possible	Probable
P13	135	216	F	44	Yes	CABG	Yes	CAD, MI	–	–	Yes	–	Possible
P14	142	192	M	41	Yes	PCI	Yes	MI	–	–	–	–	Possible
P15	204	277	F	46	Yes	–	Yes	CAD, MI	Yes	–	–	Definite	Probable

*CAD, coronary artery disease; CABG, coronary artery bypass grafting; AVR, aortic valve replacement; MI, myocardial infarction; PCI, percutaneous coronary intervention.*

For the purposes of the segregation analysis, the family members of some patients (if available) were involved in the study. The study protocol was approved by the Ethics Committee of Rajaie Cardiovascular Medical and Research Center (RHC.AC.IR.REC.1396.62), and it was conducted in accordance with the Helsinki Declaration.

### DNA Extraction

Peripheral blood samples (3–5 mL) were collected from the patients and the available family members in EDTA tubes for DNA extraction. Genomic DNA was isolated from peripheral blood using the Exgene Blood SV Mini Kit (GeneAll Seoul, South Korea). The quantity of the extracted DNA was measured with the NanoDrop Spectrophotometer (Thermo Fisher Scientific, United States).

### Polymerase Chain Reaction and Sanger Sequencing

For the amplification of the whole coding exons, exon–intron junctions, and the main parts of the introns of the *LDLR* (NG_009060) and *PCSK9* (NG_009061) genes, specific oligonucleotides were designed (Supplementary Table 1).

The forward primer 5′-AGCCTCACCTCTTACTTTTCC ATT-3′ and the reverse primer 5′-CTTTGCTTGTATGTTCT CCGTTGGT-3′ were used to amplify exon 26 of the *APOB* gene (307 base pairs in length), which creates the common FH-causing mutations (p.Arg3500Gln, p.Arg3500Trp, p.Arg3531Cys, and p.His3543Tyr).

The primers were designed using Gene Runner (Gene Runner 6.5.50), PerlPrimer (PerlPrimer 1.1.21), Primer3 (Primer3, 4.1.0), and OligoAnalyzer (OligoAnalyzer 3.1) software tools.

The polymerase chain reaction (PCR) test was performed according to an individual setup for each set of oligos, covering the aforementioned regions of the *LDLR*, *PCSK9*, and *APOB* genes. Sanger sequencing was done for all the PCR products using the ABI 3500 DNA Sequencer (Applied Biosystems, CA, United States).

### Results of the *in silico* Analysis

BioEdit software (BioEdit 7.2.1) was applied to analyze the sequencing outcomes. The identified variants of the nucleotides were analyzed through the UCSC Genome Browser^1^, ClinVar^2^, UCL^3^, and LOVD^4^ databases.

Additionally, SIFT^5^, PROVEAN^6^, PolyPhen-2^7^, and MutationTaster^8^ *in silico* predictive software tools were utilized to scrutinize the pathogenesis of the detected mutations.

## Results

### Clinic Data

Fifteen unrelated individuals with clinically diagnosed FH and premature CAD who met the SB and/or DLCNS criteria were recruited in the current study. According to the SB criteria, of the 15 patients, seven had definite FH and five possible FH. Based on the DLCNS criteria, of the 15 patients, nine had definite, two probable, and four possible FH (Table 1). The study population’s demographic characteristics, clinical features, and cholesterol levels are demonstrated in Table 1. Almost all the recruited patients had premature CAD unrelated to congenital heart diseases, hypertension, arrhythmias, or any other cardiovascular diseases.

### Spectrum of the Nucleotide Variants

**FIGURE 1:**
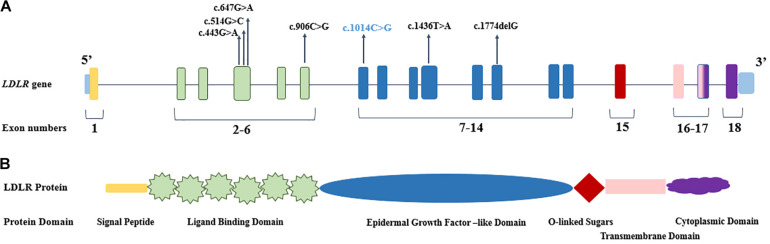
The genomic and protein organization of LDLR. **(A)** The location of identified pathogenic mutations demonstrated in the different exons of *LDLR* gene. The novel mutation presented in blue color. **(B)** The domain organization of LDLR protein, which corresponded to LDLR coding exons (with the common colors).

Direct DNA sequencing of the whole coding exons, with their flanking intron sequences, of the *LDLR* and *PCSK9* genes was performed. Also examined was the presence of the mutations (p.Arg3500Gln, p.Arg3500Trp, p.Arg3531Cys, and p.His3543Tyr) in the gene coding for *APOB*. The FH-causing variants were identified in seven (46.6%) individuals of the 15 screened patients, whereas no mutation was detected in eight patients clinically diagnosed with FH and premature CAD. Among the identified mutations, six occurred in the *LDLR* gene, whereas both *LDLR* and *PCSK9* genes were mutated in one patient (P1). The seven detected mutations were harbored in five different exons (4, 6, 7, 10, and 12) of the *LDLR* gene (Table 2 and Figure 1), and a p.Ala53Val mutation in the *PCSK9* gene was located in exon 1. The bioinformatics analysis of all the identified mutations in the *LDLR* gene categorized them as deleterious, probably damaging, and disease causing (Table 2).

**TABLE 2:** The pathogenic mutations of *LDLR* and *PCSK9* genes detected in this study.

patient ID	Mutation variants	Protein changes	Genotype	Exon	RS	Pathogeni-city	Country distribution	SIFT	Provean	Polyphen2	Mutation-Taster	1000G (allele carriers)	gnomAD	Iranome	UCL (mutation table)	LOVD-LDLR
**LDLR**																
P1	c.514G > C	p.Asp172-His	CC	4	rs8792-54554	Likely pathogenic	South Africa (Thiart et al., 2000)	Deleterious	Deleterious	Probably Damaging	Disease causing	–	–	–	reported	reported
P7	c.443G > A	p.Cys148-Tyr	GA	4	rs8792-54526	Likely pathogenic	German, United Kingdom (Nauck et al., 2001; Humphries et al., 2006)	Deleterious	Deleterious	Probably Damaging	Disease causing	–	–	–	reported	reported
P10	c.647G > A	p.Cys216-Tyr	GA	4	rs8792-54611	Likely pathogenic	German, Spanish (Dedoussis et al., 2004; Meriño-Ibarra et al., 2007)	Deleterious	Deleterious	Probably Damaging	Disease causing	–	–	–	Not reported	reported
P4	c.906C > G	p.Cys302-Trp	GG	6	rs8792-54716	Likely pathogenic	Iraqi, Turkish patient (Webb et al., 1996)	Deleterious	Deleterious	Probably Damaging	Disease causing	–	–	–	reported	reported
P2	c.1014C > G	p.Cys338-Trp	CG	7	rs879-254755	Pathogenic	Novel	Deleterious	Deleterious	Probably Damaging	Disease causing	–	–	–	Not reported	Not reported
P8	c.1436T > A	p.Leu479-Gln	AA	10	rs879-254900	Likely pathogenic	United Kingdom (Heath et al., 2001) Iranian (Fairoozy et al., 2017)	Deleterious	Deleterious	Probably Damaging	Disease causing	–	–	–	Not reported	Not reported
P9	c.1774 delG	p.G593-Afs*72	delGdelG	12	–	–	Italian N.Irish (Bertolini et al., 2017)	Deleterious	Deleterious	–	Disease causing	–	–	–	reported	Not reported
**PCSK9**																
P1	c.158C > T	p.Ala53-Val	TT	1	rs115-83680	Pathogenic	South Africa (Thiart et al., 2000)	Deleterious	Neutral	Benign	Polymor-phism	405	6099	5	–	–

Among these seven probands, four (P1, P4, P8, and P9) carried the homozygous FH-causing variants and three probands (P2, P7, and P10) carried a mutation in the heterozygous status. Our data showed that all the mutations in the *LDLR* gene (p.Asp172His, p.Cys338Trp, p.Cys302Trp, p.Cys148Tyr, p.Leu479Gln, and p.Cys216Tyr) and the mutation in the *PCSK9* gene (p.Ala53Val) were of the missense type, except for 1 mutation in the *LDLR* gene (p.G593Afs^∗^72), which was nonsense. The p.Asp172His, p.Cys302Trp, p.Cys148Tyr, p.Cys216Tyr, and p.G593Afs^∗^72 mutations in the *LDLR* gene and the p.Ala53Val mutation in the *PCSK9* gene have been reported in populations other than Iranians, whereas the p.Leu479Gln change has been reported previously in an Iranian patient. Segregation analyses were done on the available family members of the study population.

**FIGURE 2:**
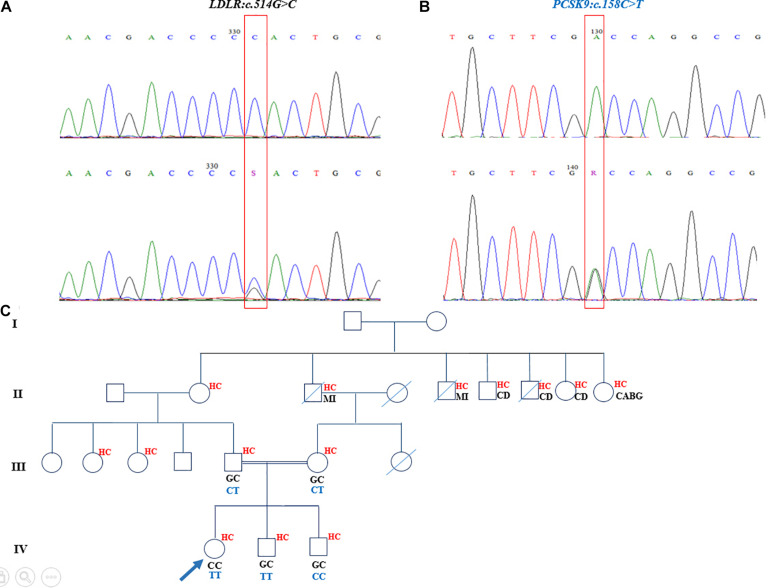
Sequencing analysis and pedigree of proband who had double mutations in *LDLR* and *PCSK9* genes. **(A)** The homozygote and heterozygote nucleotide changes at c.514G > C in *LDLR* demonstrated in upper chromaogram and lower chromatogram, respectively. **(B)** The loss-of-function mutation of *PCSK9* in homozygote and heterozygote states depicted correspondingly in upper panel and lower panel. **(C)** The genotype of proband and her family members for *LDLR* and *PCSK9* nucleotide changes demonstrated, respectively, in black and dark blue in the pedigree. The family history of proband exhibited positive history of hypercholesterolemia and cardiovascular diseases and myocardial infarction. The mother and father of the proband are first cousins. MI, myocardial infarction; HC, hypercholesterolemia; CD, cardiovascular diseases; CABG, coronary artery bypass grafting.

The homozygous nucleotide variants (c.514G > C [p.Asp172His]), harbored in exon 4 of the *LDLR* gene, and c.158C > T [p.Ala53Val], located in exon 1 of the *PCSK9* gene) were identified in an 11-year-old girl, who had undergone heart valve replacement and had high levels of TC (417 mg/dL), LDL-C (240 mg/dL), and xanthomas, with the latter seen in her knees and elbows and between her fingers. The segregation analysis confirmed that these mutations were disease-causing variants and presented in the proband’s parents and two brothers. Both of her parents and both of her siblings were heterozygous for the *LDLR* mutation, although they had different statuses vis-à-vis the *PCSK9* mutation. The c.158C > T *PCSK9* variant was in a heterozygous form in both parents but was inherited in the homozygous status in the elder brother. The younger brother, 2 years of age, was a wild type for this nucleotide change (Figure 2). The parents and the siblings had elevated cholesterol levels as well (father: TC = 306 mg/dL and LDL-C = 228 mg/dL; mother: TC = 245 mg/dL and LDL-C = 187 mg/dL; elder brother: TC = 223 mg/dL and LDL-C = 191 mg/dL; and younger brother: TC = 233 mg/dL and LDL-C = 177.8 mg/dL). The mother and father of the proband were first cousins, and there was a positive family history of myocardial infarction, cardiovascular diseases, and hypercholesterolemia in this pedigree (Figure 2). The recognized *PCSK9* mutation in this family was reported as a loss-of-function mutation.

Another homozygous mutation (c.1436T > A) in exon 10 of the *LDLR* gene was found in a 24-year-old man (P8). The patient had undergone coronary artery bypass graft (CABG) surgery and valve replacement and had a high, uncontrollable cholesterol level (TC = 461 mg/dL and LDL-C = 391 mg/dL) with detectable xanthomas. This nucleotide variant changes leucine in position 479 to glutamine, which is deleterious, probably damaging, and disease causing according to different *in silico* programs. The patient’s parents had a consanguineous marriage and a positive history of myocardial infarction, CABG, and hypercholesterolemia. The cosegregation analysis confirmed that this variant was an FH-causing mutation in the family. The proband’s father, mother, and brother were heterozygous for the mutation, whereas his sister was a wild type (Supplementary Figure 1).

A nonsense homozygous nucleotide variant (c.1774delG [p.G593Afs^∗^72]) was identified in a 22-year-old man (Supplementary Figure 2). This variant, harbored in exon 12 of the *LDLR* gene, was a frameshift mutation, where the deletion of G led to a shift in the reading frame, created a stop codon 72 amino acids downstream, and produced a truncated protein. The DNA sequencing of the *LDLR* gene demonstrated five G nucleotides in positions 1774–1778, making two glycines at a protein level (592-593 amino acids). Nonetheless, the deletion of G in any of the aforementioned positions altered the second glycine to alanine and generated stop codon 72 amino acids downstream. The software tools of SIFT and MutationTaster predicted this variant to be deleterious and disease causing, respectively. The pedigree analysis of this proband revealed a consanguineous marriage in the family. His mother and father were first cousins with a positive paternal family history of hypercholesterolemia and myocardial infarction. The proband had two younger siblings, who were apparently not affected. The father, who was the only available individual from the family, was heterozygous for this deletion. As the proband’s parents were first cousins, and both he and his father were correspondingly homozygous and heterozygous for the variant, the mother of the family, who had hypercholesterolemia, was probably heterozygous for this variant as well.

Additionally, 34 nucleotide variants, dedicated to the exons, introns, and 3-prime untranslated region (3′-UTR) of the *LDLR* gene, were detected in the 15 examined patients (Supplementary Table 2). All the nucleotide variants previously described were categorized as benign, likely benign, and with other allele. Moreover, 31 nucleotide variants were identified in the *PCSK9* gene; most of them were categorized as benign, likely benign, with other allele, and with uncertain significance allele (Supplementary Table 3). Among them, one exonic (c.1233G > A), one intronic (c.1863+20C > G), and two 3′-UTR nucleotide variants (c.^∗^863 A > G and c.^∗^980 A > G) have not been previously reported. None of the mutations of the *APOB* gene (p.Arg3500Gln, p.Arg3500Trp, p.Arg3531Cys, and p.His3543Tyr) was detected in our 15 screened patients.

### Novel Familial Hypercholesterolemia-Causing Variant

**FIGURE 3:**
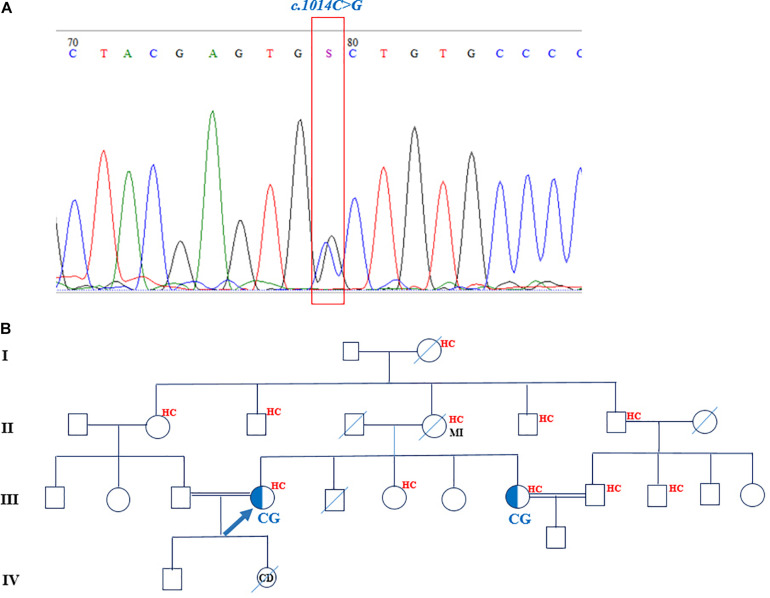
The chromatogram and pedigree of patient carried the novel mutation in *LDLR* gene. **(A)** The identified c.1014C > G nucleotide change shown in a chromatogram. **(B)** The pedigree of proband demonstrated the positive family history of hypercholesterolemia and myocardial infarction. The sister of proband is also heterozygote for the mutation. MI, myocardial infarction; HC, hypercholesterolemia.

One novel nucleotide variant (c.1014C > G) was detected and cosegregated in the available family members as well. The *in silico* mutation prediction tools of SIFT, PROVEAN, PolyPhen-2, and MutationTaster were applied to predict the functionality of the aforementioned variant. The c.1014C > G heterozygous missense mutation was found in a 39-year-old woman. This mutation corresponded to exon 7 of the *LDLR* gene and changed cysteine (C) amino acid in position 338 to tryptophan (W). SIFT, PROVEAN, PolyPhen-2, and MutationTaster predicted this variant to be deleterious, probably damaging, and disease causing, respectively. The family pedigree of the proband featured a positive history of hypercholesterolemia and myocardial infarction on the maternal side (Figure 3). The patient had three sisters and a deceased brother. Two younger sisters, aged 36 and 33 years, suffered from hypercholesterolemia as well; the youngest sister, however, was seemingly normal. The only available individual from the family was the proband’s 36-year-old sister, who was also heterozygous for this mutation. The mother, who also had hypercholesterolemia, passed away at the age of 60 years due to myocardial infarction. She probably carried the mutation. The proband and her sister, who carried the heterozygous mutation, had consanguineous marriages with their first cousins.

## Discussion

Frequent *LDLR*, *APOB*, and *PCSK9* gene mutations have been described in patients suffering from hypercholesterolemia; however, genetic data on the Iranian population are still limited (Jensen et al., 1996; Fardesfahani et al., 2005; Fardesfahani and Khatami, 2010; Farrokhi et al., 2011; Fairoozy et al., 2017; Ekrami et al., 2018; Nikkhooy et al., 2018; Tajamolian et al., 2018).

In this study, we found eight different mutations among 15 patients with clinically diagnosed FH and premature CAD. Among the study population, one patient had double mutations in both *LDLR* and *PCSK9* genes. The present study is the first investigation to report the c.1014C > G mutation. With the exception of the c.1436T > A mutation, which has been previously reported in Iran (Fairoozy et al., 2017), all the other mutations have already been reported in populations other than Iranians. Among seven mutations in the *LDLR* gene, only four nucleotide variants are reported in the UCL-FH database (Table 2), and none of the pathogenic mutations can be found in 1000Genome, gnomAD, and Iranome databases (Table 2).

Previous research on the Iranian population (Jensen et al., 1996; Fardesfahani et al., 2005; Fardesfahani and Khatami, 2010; Khan et al., 2011; Fairoozy et al., 2017; Ekrami et al., 2018; Nikkhooy et al., 2018; Tajamolian et al., 2018) has identified 15 different mutations harbored in exons 3, 4, 9, 10, 11, 12, 14, and 17 of the *LDLR* gene (Supplementary Table 4). In 2017, an investigation drawing upon target next-generation sequencing identified seven different mutations (C.389C > G, c.1436T > A, c.1599G > A, c.1729T > C, c.2001_2002delinsGT, and c.2146dupG) that were not previously reported in the Iranian population (Fairoozy et al., 2017). Two different mutations (c.285C > G and c.415G > C) were found in another study conducted on a sample of the Iranian population (Ekrami et al., 2018). The c.445G > T, c. 1246C > T, c.1478-1479delCT, c.660-661InsCC, c.105A > T, and c.1474G > A mutations have also been reported in different studies (Fardesfahani et al., 2005; Fardesfahani and Khatami, 2010; Khan et al., 2011; Nikkhooy et al., 2018; Tajamolian et al., 2018).

The detected mutations in the *LDLR* gene in the present study were scattered over exons 4, 6, 7, 10, and 12, and they encoded the functional domain, the ligand-binding domain, and the epidermal growth factor (EGF)–like of the mature LDLR protein (Figure 1). The prevalence of the identified mutations was uppermost in exon 4 (3/7 = 43%) of the *LDLR* gene (Heath et al., 2001; Villéger et al., 2002). Exon 4, along with exons 5 to 7, of the *LDLR* gene encodes the ligand-binding domain of the LDLR protein; nevertheless, exon 4 encodes the critical ligand-binding section of the protein, and any changes in the sequence may affect the folding of the protein and change the functionality of the mature protein. The consequences of missense mutations in this exon may lessen the receptor affinity of LDLR toward both APOE and APOB (Esser et al., 1988). Our data revealed c.514C > G, c.443G > A, and c.647G > A mutations in exon 4; none of these mutations has been previously reported among Iranians. Based on the previously detected mutations in the Iranian population, 5 of the 15 (33.3%) identified mutations occurred in exon 4 of *LDLR* (Supplementary Table 4). Accumulating evidence has demonstrated that individuals carrying mutations in exon 4 have a more severe FH phenotype and congenital heart disease risks; mutations in this region are therefore predominantly deleterious to receptor function (Gudnason et al., 1994; Graham et al., 1999; Humphries et al., 2006). The c.443G > A and c.647G > A mutations have both been described in Germany, the United Kingdom, and Spain (Nauck et al., 2001; Dedoussis et al., 2004; Humphries et al., 2006; Meriño-Ibarra et al., 2007).

The genetic analysis on an 11-year-old girl from the southern Iranian city of Kerman demonstrated a homozygous loss-of-function mutation in the *PCSK9* gene (c.158C > T [p.Ala53Val]) and a pathogenic homozygous mutation in the *LDLR* gene (c.514C > G [p.Asp172His]). The c.158C > T variant in *PCSK9* has also been previously reported in South Africa (Thiart et al., 2000). Several studies have revealed that loss-of-function mutations in *PCSK9* culminate in low LDL-C levels and lead to a diminished risk of CAD (Benjannet et al., 2012; Seidah, 2017; Bayona et al., 2020). Apropos the effect of loss-of-function *PCSK9* variants, some clinical trials have also confirmed that PCSK9 inhibitors lower LDL-C concentrations in the serum (Cohen et al., 2006; Stein et al., 2014; Vuorio et al., 2016; Naeli et al., 2017). Although the index patient carried the homozygous loss-of-function mutation in *PCSK9*, she presented severe phenotypes such as an uncontrollable cholesterol level; several elbows, knees, and hip xanthomas; and aortic valve disease, which necessitated valve replacement surgery. The reason for the patient’s presentations may have been completely defective *LDLR* activity owing to homozygous mutations. In addition, she was the offspring of a consanguineous marriage between a couple who presented with moderate hypercholesterolemia but had no cardiovascular diseases. The parents carried both mutations in *LDLR* and *PCSK9* in heterozygous forms. Thus, it seems that the loss-of-function mutation in *PCSK9* has a protective effect against the development of the severe forms of phenotypes (Seidah, 2019) in such individuals, notwithstanding the heterozygous mutation in *LDLR*. The proband had two brothers, who also carried the heterozygous mutation in the *LDLR* gene. In regard to the *PCSK9* gene, the elder brother, similar to the parents, featured a heterozygous mutation, whereas the younger one was a wild type.

To the best of our knowledge, this is the first report on the coincidental inheritance of a pathologic mutation in *LDLR* (p.Asp172His) and a loss-of-function mutation (p.Ala53Val) in *PCSK9* in an Iranian family. The loss-of-function mutation (c.137G > T [p.Arg46Leu]) in *PCSK9* and a pathologic mutation in *LDLR* (c.902A > G [p.Asp301Gly]), both in the heterozygous form, have been previously reported in a Caucasian family (Bayona et al., 2020).

The c.906C > G FH-causing variant (p.Cys302Trp), which was found in this study in exon 6 of *LDLR*, has also been previously identified in an Iraqi–Turkish patient (Webb et al., 1996). The 40-year-old woman carrying this mutation originated from the north of Iran. Given the close geographical and cultural links between Iraq, Turkey, and Iran, it is probable that the two patients shared some common founder mutations.

The novel mutation found in this study (c.1014C > G), which is located in exon 7 of the *LDLR* gene, changes cysteine to tryptophan. According to the LOVD database, approximately 3% of the total 3731 variants of *LDLR* happen on exon 7 (Fokkema et al., 2005). This variant has not been previously identified; still, the same location features another change that has been previously reported (c.1014C > A). This variation, which is a stop-gained mutation, alters cysteine in position 338 to the termination code and creates a truncated protein (Kotzer and Baudhuin, 2009). Exon 7, along with exons 8–12, of the *LDLR* gene participates in the production of the EGF precursor homology domain of LDLR. This domain of LDLR shares such conserved sequences as cysteine-rich sequences, YWTD repeats, and EGF repeats to EGF (Davis et al., 1987; Springer, 1998). Furthermore, the EGF precursor-like domain comprised two EGF homology domains (EGF-A and EGF-B), dispersed from the third EGF-like domain by a B-propeller domain. Exon 7 of *LDLR* encodes the EGF-A part of the EGF-like domain of LDLR, which encompasses 40-amino-acid residues and six cysteine amino acids, forming three disulfide bonds (Springer, 1998; Jeon et al., 2001). In the molecular mechanisms of LDLR recycling in hepatocytes, PCSK9 binds to the EGF-A domain of LDLR in a PH- and calcium-dependent manner and through lysosomal degradation decreases the total LDLR level in the liver (Zhang et al., 2007; Bottomley et al., 2009). A deficiency in this region interferes with the release of the binding ligand of the internalized LDLR and inhibits its recycling to the cell surface (Davis et al., 1987). On account of the fact that this missense mutation exists in a cysteine residue, it may result in misfolding and defective protein receptors. In addition, all *in silico* predictive programs describe this variant as deleterious, probably damaging, and disease causing. The segregation analysis of this variant in the index patient’s sister, who had hypercholesterolemia, confirmed the pathogenicity of this variant as well.

The point mutations (p.leu479Gln and p.G593Afs^∗^72) were respectively harbored in exons 10 and 12, which encode different parts of the LDLR EGF precursor-like domain. Exon 10 encodes part of a 5-repeat region of 40–60 amino acids that has a conserved motif (YWTD). This part is also known to play a role in the intracellular trafficking and recycling of LDLR, which explains why mutations in this section are acknowledged to be the cause of transport-defective or recycling-defective proteins (Brown and Goldstein, 1974; Davis et al., 1987). The p.leu479Gln mutation has been previously described in Iran (Fairoozy et al., 2017) and the United Kingdom (Heath et al., 2001). Our segregation analysis of this mutation in the first degree-relatives of the proband (P8) confirmed the pathogenicity of the mutation too. The translation product of the allele carrying the frameshift mutation (p.G593Afs^∗^72) leads to the truncation of the proteins of 663 amino acids and is deemed a receptor-negative mutation (Bertolini et al., 2017). With respect to the pathogenicity of this mutation, confirmed via the molecular diagnosis of the variation in the family of the proband (P9), the result of the screening of the family demonstrated that the father carried this mutation in a heterozygous state. It is also probable that the proband’s mother was also heterozygous for this variation. This mutation was originally found in Italian and Northern Ireland populations (Bertolini et al., 2017).

Furthermore, we succeeded in detecting 31 different nucleotide variants in the *PCSK9* gene. While most of them have been previously reported, there are some unreported variants scattered in exons, introns, and 3′-UTRs (Supplementary Table 3). The UTR parts (5′ and 3′) include regulatory elements that interact with such different regulatory factors as transcription factors and microRNAs. Altering the nucleotides in the UTRs of genes may interfere with their interactions and causes the misexpression of their targeted gene (Chatterjee and Pal, 2009; Azad et al., 2020). The existing evidence notwithstanding, this hypothesis still requires more functional analyses to be confirmed.

In the present study, we detected none of the common *APOB* mutations, suggesting that the frequency of *APOB* mutations in Iran may differ from that in other countries. Further research on this point is warranted.

Among our study population, which comprised 15 subjects with clinically diagnosed FH and premature CAD, we detected eight patients who were negative for FH mutations. Accumulating evidence has shown that, in addition to mutations in the *LDLR* and *PCSK9* genes, mutations in other single or multiple genes can contribute to the creation of the FH phenotype (Smilde et al., 2001; Nordestgaard et al., 2013; Cuchel et al., 2014b; Fouchier et al., 2014; Brautbar et al., 2015; Berberich and Hegele, 2019). The accumulation of common small-effect LDL-C–raising alleles can elevate LDL-C levels and, consequently, cause FH (Talmud et al., 2013). Accordingly, the absence of mutations in the FH-mutation–negative probands in our study may be attributable to nucleotide alterations in other genes, nucleotide variations in other parts of the *APOB* gene (not addressed in this study), or a combination of LDL-C-raising alleles. Be that as it may, previous studies on other diseases have demonstrated that the presence and combinations of some polymorphisms can render individuals more susceptible to disease (Khajali and Khajali, 2014; Mesic et al., 2019; Pinheiro et al., 2019).

We succeeded in finding several nucleotide variations/polymorphisms in both *LDLR* and *PCSK9* scattered in exons, introns, and 5′–3′ UTRs. These nucleotide variations/polymorphisms could be the combination of different polymorphisms, predisposing FH-mutation–negative individuals to hypercholesterolemia.

### Study Limitations

The results of the present study should be interpreted in light of its limitations. First, our small sample size precluded an accurate estimation of the prevalence of the mutations in the Iranian population. Budget constraints also limited our investigation to the screening of only the *LDLR* and *PCSK9* genes and a part of the *APOB* gene. Indubitably, many other hitherto reported and unreported genes may be the underlying causes of FH-mutation negativity. A large insertion or deletion in the *LDLR* gene causes FH in 5–10% of patients (Hobbs et al., 1985; Goldmann et al., 2010); we were, unfortunately, unable to detect such mutations. Finally, what has thus far hampered research on polygenic FH in Iran is, aside from financial restraints, the dearth of data on the frequency of polymorphisms in the Iranian population.

## Conclusion

The existing literature contains a paucity of information regarding genetic testing on nucleotide alterations in the Iranian population. The finding of seven different mutations in this study, along with other studies on samples of the Iranian population, demonstrates the high degree of genetic heterogeneity and the wide spectrum of mutations in Iranians. This domain undoubtedly requires further in-depth research. In daily practice, patients presenting with premature CAD are rarely screened for the genetic analysis of FH; this is a missed opportunity for preventive therapy. Given that the first-degree relatives of patients with FH have a 50% chance of affliction, a targeted molecular genetic screening of individuals with premature CAD and FH is an effective strategy both to prevent cardiovascular diseases in their nascent stages and to confer more desirable management plans.

## Accession Number

The accession number of the novel and reported variants are as follows:

***LDLR***
c.1014C>G variant: [ClinVar]: [SCV001446309]
c.443G>A variant: [ClinVar]: [SCV001467722]
c.647G>A variant: [ClinVar]: [SCV001467723.1]
c.906C>G variant: [ClinVar]: [SCV001467724.1]
c.1436T>A variant: [ClinVar]: [SCV001467725.1]
c.1774delG variant: [ClinVar]: [SUB8843875, processing]
c.514G>C variant: [ClinVar]: [SUB8843863, processing]
***PCSK9***
c.1233G>A variant: [ClinVar]: [SCV001450531]
c.1863+20C>G variant: [ClinVar]: [SCV001450532]
c.^∗^863A>G variant: [ClinVar]: [SCV001450537]
c.^∗^980A>G variant: [ClinVar]: [SCV001450538]

## Data Availability Statement

The datasets presented in this study can be found in online repositories. The names of the repository/repositories and accession number(s) can be found in the article/Supplementary Material.

## Ethics Statement

The studies involving human participants were reviewed and approved by Rajaie Cardiovascular, Medical and Research Center Ethics Committees. Written informed consent to participate in this study was provided by the participants’ legal guardian/next of kin.

## Author Contributions

AM: experiment design, lab work, data production, data interpretation, and MS edit. MMale, ZG, ZK, and FN: clinical evaluation of patients, data interpretation, and MS edit. MMo: lab work, data production, and MS edit. SK: data interpretation and MS edit. SM and NS: experiment design, data interpretation, and MS edit. MMala: experiment design, data interpretation, writing the first draft of MS, and manuscript final edit. All authors contributed to the article and approved the submitted version.

## Conflict of Interest

The authors declare that the research was conducted in the absence of any commercial or financial relationships that could be construed as a potential conflict of interest.

## Footnotes

1 https://genome.ucsc.edu

2 www.ncbi.nlm.nih.gov/clinvar

3 https://www.ucl.ac.uk/fh-old/muttab.html

4 https://www.LOVD.nl/LDLR

5 https://sift.bii.a-star.edu.sg/

6 provean.jcvi.org

7 genetics.bwh.harvard.edu/pph2

8 www.mutationtaster.org

## Supplementary Material

Supplementary Figure 1 Sequencing analysis and pedigree of proband with c.1436T>A nucleotide change in *LDLR* gene. **(A)** The mutation found in proband in a homozygote form (upper chromatogram) and identified in a heterozygote statue in father of the patient (lower chromatogram). **(B)** The pedigree of the proband demonstrated the consanguineous marriage, positive family history of hypercholesterolemia, myocardial infarction and cardiovascular diseases. The genotypes of his sibling and his parent exhibited in dark blue color as well. MI, HC, and CD stand for myocardial infarction, hypercholesterolemia, and cardiovascular diseases, respectively.

Supplementary Figure 2 The chromatogram and pedigree of patient who carried the c.1774delG mutation in *LDLR* gene. **(A)** The upper chromatogram demonstrated the sequence in wild type. The homozygote deletion mutation in nucleotide 1774 exhibited in middle chromatogram and the heterozygote form depicted in lower chromatogram. **(B)** The pedigree of the proband showed the consanguineous marriage and positive family history of hypercholesterolemia and MI. MI and HC stand for Myocardial infarction and Hypercholesterolemia, respectively.

Supplementary Table 1 The sequences of all designed oligonucleotides utilized in this study to perform the PCRs and Sanger sequencing.

Supplementary Table 2 All nucleotide variations of *LDLR* gene found in this study.

Supplementary Table 3 All nucleotide variations of *PCSK9* gene found in this study.

Supplementary Table 4 The previously reported pathogenic mutations in Iranian population.
