# Supplementary figures and images for: Mutational Spectrum of LDLR and PCSK9 Genes Identified in Iranian Patients With Premature Coronary Artery Disease and Familial Hypercholesterolemia
Source: Front Genet. 2021 Feb 11;12:625959. doi: 10.3389/fgene.2021.625959 (PMC7959244; doi:10.3389/fgene.2021.625959)

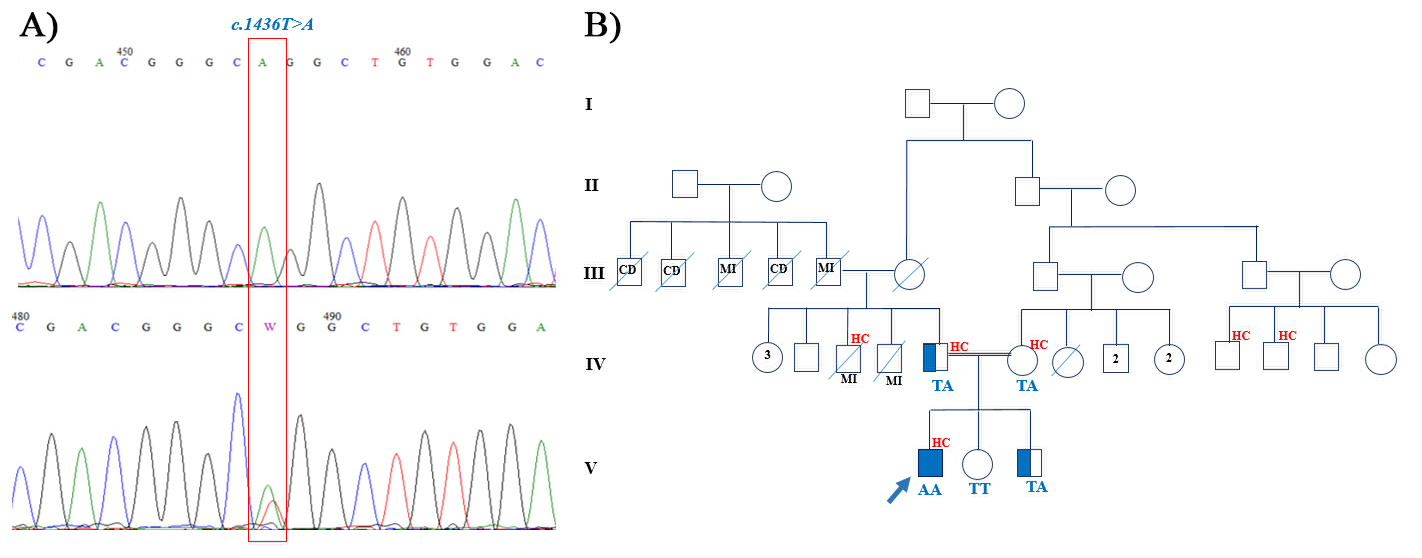

Supplement: Supplementary Figure 1 — Sequencing analysis and pedigree of proband with c.1436T>A nucleotide change in LDLR gene. (A) The mutation found in proband in a homozygote form (upper chromatogram) and identified in a heterozygote statue in father of the patient (lower chromatogram). (B) The pedigree of the proband demonstrated the consanguineous marriage, positive family history of hypercholesterolemia, myocardial infarction and cardiovascular diseases. The genotypes of his sibling and his parent exhibited in dark blue color as well. MI, HC, and CD stand for myocardial infarction, hypercholesterolemia, and cardiovascular diseases, respectively. [file Image_1.TIF]

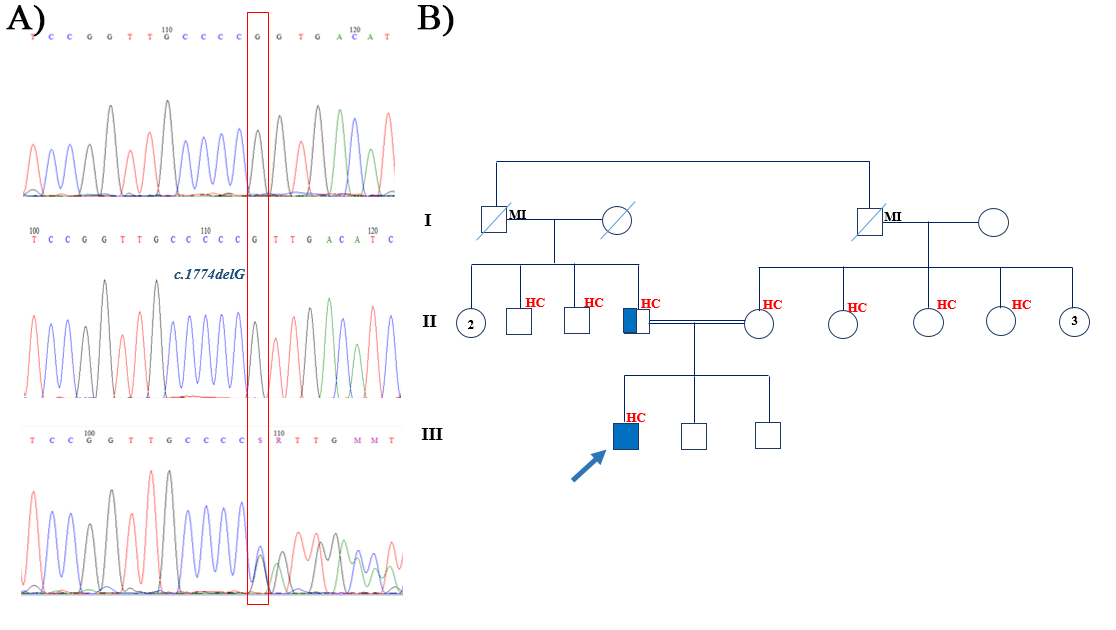

Supplement: Supplementary Figure 2 — The chromatogram and pedigree of patient who carried the c.1774delG mutation in LDLR gene. (A) The upper chromatogram demonstrated the sequence in wild type. The homozygote deletion mutation in nucleotide 1774 exhibited in middle chromatogram and the heterozygote form depicted in lower chromatogram. (B) The pedigree of the proband showed the consanguineous marriage and positive family history of hypercholesterolemia and MI. MI and HC stand for Myocardial infarction and Hypercholesterolemia, respectively. [file Image_2.TIF]
