# Supplementary material for: Mutational Spectrum of LDLR and PCSK9 Genes Identified in Iranian Patients With Premature Coronary Artery Disease and Familial Hypercholesterolemia
Source: Front Genet. 2021 Feb 11;12:625959. doi: 10.3389/fgene.2021.625959 (PMC7959244; doi:10.3389/fgene.2021.625959)
Supplement: Supplementary Table 1 — The sequences of all designed oligonucleotides utilized in this study to perform the PCRs and Sanger sequencing. [file Table_1.DOCX]

| Length | Tm | Reverse Primer | Tm | Forward Primer | ***LDLR*** |
| --- | --- | --- | --- | --- | --- |
|  |  | 5’-3’ Sequences |  | 5’-3’ Sequences | EXON |
| 550bp | 61 | GCTCCCTCTCAACCTATTCT | 60 | ATTAGGCTATTGGAGGATCTTG | 1 |
| 429bp | 60 | GAATACATCAAAATCCACTGGC | 62 | TAGTTGGCAGGAAATAGACACAG | 2 |
| 471bp | 62 | ATCCCCACTTTGTAATGCCTC | 61 | GTTTCACTATATTGGCCAGGC | 3 |
| 619bp | 64 | TAAATCACTGCATGTCCCACAC | 66 | AGAGGGCAGTGGTTCAGAGT | 4 |
| 1192bp | 64 | AACCCTACAGCACTCATGTCTC | 63 | TCTCTGGTTGTCTCTTCTTGAGAA | 5,6 |
| 1328bp | 64 | GTCAGGGGATATGAGTCTGTGC | 62 | CGAGAGTGACCAGTCTGCAT | 7,8 |
| 744bp | 63 | CTGGGATTACAGGTGCTTTGAG | 65 | CCGTTGGGAGGTCTTTTCCAC | 9,10 |
| 1135bp | 62 | TTGGCTTGAGTGATCTATAGTCTG | 62 | CAGCAGGACTATTTCCCAAGC | 11,12 |
| 661bp | 66 | GGTGCTGATGTGTCCATCCAAG | 68 | TAGTTGTGGAGAGAGGGTGGCC | 13,14 |
| 403bp | 62 | GACGACACCTGGACTCCATC | 63 | ACTCCTGGACTCACTCAAGAGAT | 15 |
| 495bp | 64 | AGCTGTCTGATCTTGTCACTGTC | 62 | TAGCCAGACCTCTGTTTCTATCC | 16 |
| 384bp | 62 | TTCACCTAATGCTGTCCTCG | 63 | GAGTCAAGGTTATGGTACGATGC | 17 |
| 746bp | 63 | GTGAATGAGTTTGGTCTCGGTG | 65 | CAGGAGGTGAGAAGTAGGTGGC | 18 |
|  |  | 5’-3’ Sequences |  | 5’-3’ Sequences | ***PCSK9*** |
|  |  |  |  |  | EXON |
| 731 bp | 64 | GCACTCCACTTCCTCTCTTACA | 65 | CGAAACCTGATCCTCCAGTCC | 1 |
| 706 bp | 59 | ATAGCAACAGCTTCAAAGGAATT | 58 | AGTAGGGGTGAGATAAAGTACA | 2 |
| 285 bp | 63 | GGCAGAGCAAATGGATTCAGC | 62 | GGATGTGGGGACAGGTTTGA | 3 |
| 750 bp | 66 | TTGGCACTCTGGTTCTCTGGCT | 65 | GCCTGGGATGTGCTCTGTAGTT | 4,5 |
| 451 bp | 62 | CTGCTATTCAGACTCTGCGATG | 64 | GGTGACCTTGGCTTTGTTCCTC | 6 |
| 385 bp | 66 | AGGGGCTGTTAGCATCACGGT | 64 | CTGGGCAGTCAGATTTTCCTTAGG | 7 |
| 850 bp | 64 | AAGAGCTGGAGTCTGGAGGATG | 62 | CGAGAAGAGAGCTTAGTGTCTGT | 8,9 |
| 566 bp | 60 | CAGTGGGTGCATAAGGAGAA | 61 | CTTGAGGAGATGGGGTCTTAATG | 10 |
| 344 bp | 66 | GCAGGAGAGACACGCAGCAC | 64 | GGTTTCCTAGCTCTTGCCTCAGA | 11 |
| 767 bp | 64 | CATCAGCACCTTTCACACTCACC | 62 | GATGTCGGAGGGAGAAATGAAGT | 12 |
|  |  | 5’-3’ Sequences |  | 5’-3’ Sequences | ***APOB*** |
| 307bp | 62 | CTTTGCTTGTATGTTCTCCGTTGGT | 60 | AGCCTCACCTCTTACTTTTCCATT | 26 |

**Supplementary Table 1**
