# Supplementary material for: Mutational Spectrum of LDLR and PCSK9 Genes Identified in Iranian Patients With Premature Coronary Artery Disease and Familial Hypercholesterolemia
Source: Front Genet. 2021 Feb 11;12:625959. doi: 10.3389/fgene.2021.625959 (PMC7959244; doi:10.3389/fgene.2021.625959)
Supplement: Supplementary Table 2 — All nucleotide variations of LDLR gene found in this study. [file Table_2.DOCX]

| **Ser. Number** | **Mutation (LDLR)** | | **Frequency in this study** | **Genotype**  **(per patients)** | | **RS** | **Location** | **Clinical Significance** |
| --- | --- | --- | --- | --- | --- | --- | --- | --- |
|  | NM_000527.4 | NP_000518.1 |  | het | hom |  |  |  |
| **Exonic** | | | | | | | | |
| 1 | c.81C>T | p.Cys27= | 4/15 | 3/4 | 1/4 | rs2228671 | 2 | Benign |
| 2 | c.1194C>T | p.Ile398= | 1/15 | 1/1 | - | rs13306498 | 9 | With other allele |
| 3 | c.1413A>G | p.Arg471= | 10/15 | 4/10 | 6/10 | rs5930 | 10 | With other allele |
| 4 | c.1617C>T | p.Pro539= | 1/15 | 1/1 | - | rs5929 | 11 | With other allele |
| 5 | c.1773C>T | p.Asn591= | 6/15 | 2/6 | 4/6 | rs688 | 12 | Benign |
| 6 | c.1725C>T | p.Leu575= | 2/15 | 1/2 | 1/2 | rs1799898 | 12 | Benign |
| 7 | c.1774G>A | p.Gly592Arg | 1/15 | - | 1/1 | rs763147599 | 12 | With other allele |
| 8 | c.2232A>G | p.Arg744= | 12/15 | 2/12 | 10/12 | rs5927 | 15 | Benign |
| **Intronic** | | | | | | | | |
| 9 | c.190+56G>A | - | 2/15 | 2/2 | - | rs3745677 | 2 | With Likely benign allele |
| 10 | c.314-50T>C | - | 2/15 | 2/2 | - | rs10423288 | 3 | With Benign allele |
| 11 | c.1060+8C>T | - | 4/15 | - | 4/4 | rs1391461586 | 7 | NA |
| 12 | c.1060+10G>C | - | 8/15 | 4/8 | 4/8 | rs12710260 | 7 | With other allele |
| 13 | c.1060+7T>C | - | 4/15 | - | 4/4 | rs2738442 | 7 | With other allele, |
| 14 | c.1060+86C>G | - | 1/15 | 1/1 | - | rs761306775 | 7 | NA |
| 15 | c.1359-30C>T | - | 9/15 | 5/9 | 4/9 | rs1003723 | 9 | With Benign allele |
| 16 | c.1586+53A>G | - | 3/15 | 1/3 | 2/3 | rs1569372 | 10 | NA |
| 17 | c.1705+182C>T | - | 10/15 | 4/10 | 6/10 | rs2738445 | 11 | NA |
| 18 | c.1706-209C>A | - | 12/15 | 4/12 | 8/12 | rs2738446 | 11 | NA |
| 19 | c.1706-55A>C | - | 9/15 | 3/9 | 6/9 | rs2738447 | 11 | With Benign allele |
| 20 | c.1705+56C>T | - | 6/15 | 3/6 | 3/6 | rs4508523 | 11 | With Likely benign allele |
| 21 | c.1705+209C>T | - | 6/15 | 2/6 | 4/6 | rs7248981 | 11 | NA |
| 22 | c.1706-69G>T | - | 6/15 | 2/6 | 4/6 | rs7259278 | 11 | With Benign allele |
| 23 | c.2141-18G>A | - | 1/15 | 1/1 | - | rs1374712856 | 14 | NA |
| 24 | c.2312-192A>G | - | 4/15 | 1/4 | 3/4 | rs12459476 | 15 | NA |
| 25 | c.2312-136A>G | - | 9/15 | 1/9 | 8/9 | rs2569538 | 15 | With Likely benign allele |
| 26 | c.2312-211A>C | - | 5/15 | - | 5/5 | rs2738459 | 15 | NA |
| 27 | c.2312-47G>A | - | 1/15 | 1/1 | - | rs41306974 | 15 | With Benign allele |
| 28 | c.2389+46C>T | - | 5/15 | 3/5 | 2/5 | rs2738460 | 16 | With Benign allele |
| 29 | c.2389+47G>A | - | 1/15 | 1/1 | - | rs13306501 | 16 | With Benign allele |
| **3'-UTR** | | | | | | | | |
| 30 | c.*52G>A | - | 7/15 | 4/7 | 3/7 | rs14158 | 3' | With other allele |
| 31 | c.*315G>C | - | 13/15 | 3/13 | 10/13 | rs2738464 | 3' | With Likely benign allele |
| 32 | c.*504G>A | - | 10/15 | 1/10 | 9/10 | rs2738465 | 3' | With Likely benign allele |
| 33 | c.*141G>A | - | 3/15 | 3/3 | - | rs3826810 | 3' | With Likely benign allele |
| 34 | c.*223G>A | - | 1/15 | 1/1 | - | rs17243011 | 3' | NA |

**Supplementary Table 2**
