# Supplementary material for: Mutational Spectrum of LDLR and PCSK9 Genes Identified in Iranian Patients With Premature Coronary Artery Disease and Familial Hypercholesterolemia
Source: Front Genet. 2021 Feb 11;12:625959. doi: 10.3389/fgene.2021.625959 (PMC7959244; doi:10.3389/fgene.2021.625959)
Supplement: Supplementary Table 3 — All nucleotide variations of PCSK9 gene found in this study. [file Table_3.DOCX]

| **Ser. Number** | **Mutation (PCSK9)** | | **Frequency** | **Genotype**  **(per patients)** | | **RS** | **Location** | **Clinical Significance** |
| --- | --- | --- | --- | --- | --- | --- | --- | --- |
|  | NM_174936.3 | NP_777596.2 |  | het | hom |  |  |  |
| **5'UTR** | | | | | | | | |
| 1 | c.-64C>T | - | 1/15 | - | 1/1 | rs45448095 | 5**'** | With other allele |
| **Exonic** | | | | | | | | |
| 2 | c.43_45delCTG | p.Leu23del | 3/15 | 1 | 2/2 | rs751675284 | 1 | NA |
| 3 | c.1026A>G | p.Gln342= | 15/15 | - | 15/15 | rs509504 | 7 | With other allele |
| 4 | c.1233G>A | p.Leu411= | 6/15 | 6/6 | - | - | 8 | Not reported |
| 5 | c.1327G>A | p.Ala443Thr | 1/15 | 1/1 | - | rs28362263 | 8 | With other allele |
| 6 | c.1380A>G | p.Val460= | 15/15 | 5/15 | 10/15 | rs540796 | 9 | With Benign allele |
| 7 | c.1420G>A | p.Val474Ile | 15/15 | 5/15 | 10/15 | rs562556 | 9 | With Benign allele |
| 8 | c.2009G>A | p.Gly670Glu | 14/15 | - | 14/14 | rs505151 | 12 | With other allele |
| **Intronic** | | | | | | | | |
| 9 | c.207+15A>G | - | 15/15 | 3/15 | 12/15 | rs2495482 | 1 | With other allele |
| 10 | c.399+165T>C | - | 5/15 | 3/5 | 2/5 | rs4927193 | 2 | NA |
| 11 | c.399+308C>T | - | 1/15 | 1/1 | - | rs74700387 | 2 | NA |
| 12 | c.399+193C>T | - | 1/15 | 1/1 | - | rs2479411 | 2 | NA |
| 13 | c.399+232T>C | - | 1/15 | 1/1 | - | rs4275490 | 2 | NA |
| 14 | c.657+82G>A | - | 14/15 | 9/14 | 5/14 | rs625619 | 4 | With Benign allele |
| 15 | c.657+114delC | - | 10/15 | 5/10 | 5/10 | rs397735050 | 4 | With Uncertain significance allele |
| 16 | c.658-7C>T | - | 10/15 | 4/10 | 6/10 | rs2483205 | 4 | With other allele |
| 17 | c.657+113C>A | - | 1/15 | 1/1 | - | rs536037529 | 4 | NA |
| 18 | c.657+9G>A | - | 3/15 | 3/3 | - | rs11800243 | 4 | With other allele |
| 19 | c.657+76C>A | - | 3/15 | 3/3 | - | rs11806638 | 4 | With Benign allele |
| 20 | c.657+114C>A | - | 2/15 | 2/2 | - | rs7552350 | 4 | With Benign allele |
| 21 | c.658-36G>A | - | 3/15 | 3/3 | - | rs11800265 | 4 | With Uncertain significance allele |
| 22 | c.799+3A>G | - | 15/15 | 4/15 | 11/15 | rs2495477 | 5 | With other allele |
| 23 | c.799+64C>A | - | 10/15 | 7/10 | 3/10 | rs494198 | 5 | With Benign allele |
| 24 | c.996+39_996+44delGGCGGA | - | 14/15 | 11/14 | 3/14 | rs1370704687 | 6 | NA |
| 25 | c.1354+102C>T | - | 9/15 | 2/9 | 7/9 | rs584626 | 8 | NA |
| 26 | c.1355-56C>T | - | 15/15 | 6/15 | 9/15 | rs585131 | 8 | With Benign allele |
| 27 | c.1863+20C>G | - | 12/15 | 12/12 | - | - | 11 | Not reported |
| **3'UTR** | | | | | | | | |
| 28 | c.*571C>T | - | 15/15 | 8/15 | 7/15 | rs662145 | 3' | With Likely benign allele |
| 29 | c.*863 A>G | - | 2/15 | 2/2 | - | - | 3' | Not reported |
| 30 | c.*980 A>G | - | 3/15 | 3/3 | - | - | 3' | Not reported |
| 31 | c.*849T>G | - | 1/15 | 1/1 | - | rs28362292 | 3' | With Likely benign allele |

**Supplementary Table 3**
