# Supplementary material for: Mutational Spectrum of LDLR and PCSK9 Genes Identified in Iranian Patients With Premature Coronary Artery Disease and Familial Hypercholesterolemia
Source: Front Genet. 2021 Feb 11;12:625959. doi: 10.3389/fgene.2021.625959 (PMC7959244; doi:10.3389/fgene.2021.625959)
Supplement: Supplementary Table 4 — The previously reported pathogenic mutations in Iranian population. [file Table_4.DOCX]

| Mutations | | Exons | Reported Country | Reference |
| --- | --- | --- | --- | --- |
| [NM_000527.4](http://www.ncbi.nlm.nih.gov/nuccore/307775410) | [NP_000518.1](http://www.ncbi.nlm.nih.gov/protein/4504975) |  |  |  |
| c.285C>G | Cys95Trp | 3 | Iran | (Ekrami et al., 2018) |
| c.345G>T | Arg115Arg | 3 | Iran | (Fardesfahani and Khatami, 2010) |
| 445G>T | Gly149Cys | 4 | Iran, Malaysian | (Fardesfahani et al., 2005; Al-Khateeb et al., 2011) |
| c.415G>C | Asp139His | 4 | Iran | (Ekrami et al., 2018) |
| c.660-661InsCC | - | 4 | Iran | (Fardesfahani and Khatami, 2010) |
| c.105A>T | Gln35His | 4 | Iran | (Fardesfahani and Khatami, 2010) |
| C.389C>G | Ser130* | 4 | Denmark, Iran | (Damgaard et al., 2005; Fairoozy et al., 2017) |
| c. 1246C>T | Arg416Trp | 9 | Iran, Spanish | (García‐García et al., 2001; Tajamolian et al., 2018) |
| c.1478-1479delCT | Ser493Cfs*41 | 10 | Iran, [German](https://www.ucl.ac.uk/fh-old/ethnic.html#17) , [Italian](https://www.ucl.ac.uk/fh-old/ethnic.html#1), [Austrian](https://www.ucl.ac.uk/fh-old/ethnic.html#26) | (Cavanaugh et al., 1994; Jensen et al., 1996; Raal et al., 1998; Fellin et al., 2015) |
| c.1436T>A | Leu479Gln | 10 | Iran | (Fairoozy et al., 2017) |
| c.1474G>A | Asp492Asp | 10 | Iran, [Austrian](https://www.ucl.ac.uk/fh-old/ethnic.html#26) ,[Chinese (Hong Kong)](https://www.ucl.ac.uk/fh-old/ethnic.html#52) | (Varret et al., 1998; Little et al., 2002; Nikkhooy et al., 2018) |
| c.1599G>A | Trp533* | 11 | Iran, Netherland, Portuguese, Japanese | (Hattori et al., 1999; Fouchier et al., 2005; Medeiros et al., 2010; Vandrovcova et al., 2013; Fairoozy et al., 2017) |
| c.1729T>C | Trp577Arg | 12 | Turkey, Iran | (Sözen et al., 2005; Schmidt et al., 2008; Fairoozy et al., 2017) |
| c.2001_2002delinsGT | Cys667Trp | 14 | Iran, France | (Nissen et al., 1998; Vandrovcova et al., 2013; Fairoozy et al., 2017) |
|  | Glu668* |  |  |  |
| c.2146dupG | Val806Glyfs*11 | 17 | Iran, US, Sweden, Czech, Netherlands, Japan | (Ekström et al., 1998; Nobe et al., 1999; Fouchier et al., 2001; Kuhrová et al., 2002; Yu et al., 2002; Fairoozy et al., 2017) |

**Supplementary Table 4**
